# Janus Particles at Fluid Interfaces: Stability and Interfacial Rheology

**DOI:** 10.3390/nano11020374

**Published:** 2021-02-02

**Authors:** Elton L. Correia, Nick Brown, Sepideh Razavi

**Affiliations:** School of Chemical, Biological, and Materials Engineering, University of Oklahoma, 100 E. Boyd Street, Norman, OK 73019, USA; correiaelton@ou.edu (E.L.C.); nicholas.m.brown-1@ou.edu (N.B.)

**Keywords:** Janus particles, fluid interfaces, interfacial rheology, Pickering emulsions and foams

## Abstract

The use of the Janus motif in colloidal particles, i.e., anisotropic surface properties on opposite faces, has gained significant attention in the bottom-up assembly of novel functional structures, design of active nanomotors, biological sensing and imaging, and polymer blend compatibilization. This review is focused on the behavior of Janus particles in interfacial systems, such as particle-stabilized (i.e., Pickering) emulsions and foams, where stabilization is achieved through the binding of particles to fluid interfaces. In many such applications, the interface could be subjected to deformations, producing compression and shear stresses. Besides the physicochemical properties of the particle, their behavior under flow will also impact the performance of the resulting system. This review article provides a synopsis of interfacial stability and rheology in particle-laden interfaces to highlight the role of the Janus motif, and how particle anisotropy affects interfacial mechanics.

## 1. Introduction

The behavior of colloidal particles in vicinity of fluid interfaces has intrigued scientists ever since particles were first observed to reside at the surface of droplets and bubbles yielding stabilization in emulsions and foams [1,2]. There is a vast range of applications in which particles are used in engineering the performance of interfacial systems including pharmaceutics, food industry, oil recovery, and personal care products [3,4,5,6,7,8,9,10]. By binding to fluid interfaces, particles remove the energetically unfavorable contact area between the two fluids and replace it with solid/fluid interfaces, which results in an overall reduction in the free energy of the system. The energy required to desorb an interfacially-trapped particle thus depends on the interfacial tension of the fluids, particle contact angle at the interface, and particle size. A large value of binding energy relative to the thermal energy can therefore lead to irreversibly adsorbed particles at the interface [11,12,13,14,15,16,17]. As such, parameters including particle size, wettability, and concentration have been used to alter the stability of emulsions and foams [18,19,20,21,22,23,24,25,26,27,28,29,30,31,32,33]. With the recent advancements in synthesis and fabrication techniques, particle anisotropy (both in shape and surface properties) has been introduced as another avenue for manipulating the behavior of particles at fluid interfaces. Not only this is a significant step from the standpoint of fundamental science, but is also essential from the practical point of view where in many real applications the particles possess heterogeneities and non-idealities [34,35,36,37,38,39,40]. Therefore, due to these deviations, their behavior cannot be fully described by our understanding of homogeneous particles.

**Figure 1:**
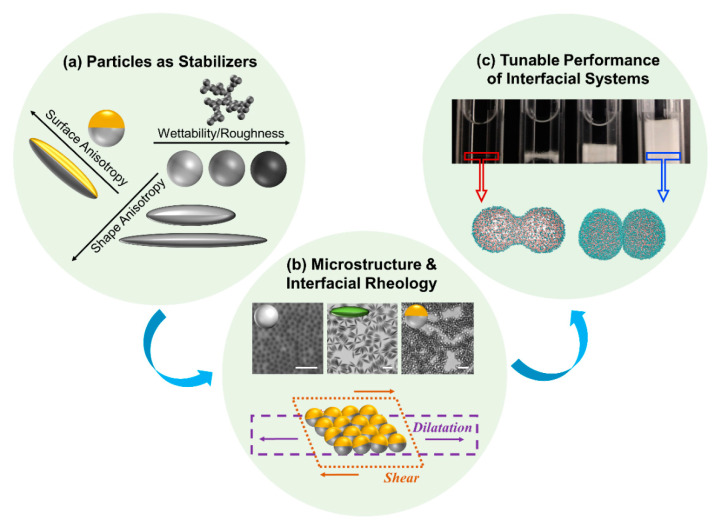
(Color online) Particle-stabilized interfacial systems: (**a**) schematics of particle attributes affecting their microstructure at a fluid interface; (**b**) different microstructures resulting from spherical homogeneous particles vs. ellipsoidal homogeneous particles vs. spherical Janus particles at the air/water interface (scale bar is 5 μm); representation of both interface deformation modes imposed on the interface, i.e., dilatation and shear; (**c**) rectangular test tubes (2 by 8 mm polycarbonate cells) containing Pickering emulsions with different degrees of stability along with Dissipative Particle Dynamics simulations of the coalescence of emulsion droplets (droplet diameter 27 nm) [105].

The focus of this review paper is so-called Janus particles—named after a two-faced Roman God—and their behavior at fluid interfaces. This term is applied to particles with a dual characteristic that possess anisotropic surface properties where one face of the particle has one chemistry and the other has another chemistry yielding an amphiphilic character, shown in Figure 1a [41,42,43,44,45]. In addition to surface chemical anisotropy, the particles can also be shape anisotropic [46,47]. The “Janus” motif, first fabricated by Casagrande et al. [48] and later highlighted by the Nobel Laureate Pierre-Gilles de Gennes in 1991, has impacted several fields including the bottom-up assembly of novel functional structures using Janus colloidal building blocks [49,50,51,52,53,54,55], active nanomotors [56,57,58,59,60,61,62,63], biological imaging and sensing [64,65], drug delivery [66,67,68,69,70], and tunable stability in emulsions, foams and polymer blends [71,72,73,74,75,76,77]. The interfacial applications are of particular interest [78,79,80,81] since Janus particles combine the colloidal-scale properties of particle stabilizers (i.e., large energy of desorption from fluid interfaces) [82,83] with the molecular-scale properties of surfactants (i.e., amphiphilicity and reduction of interfacial tension) [78,84,85,86,87,88]. For example, Glaser et al. reported that homogenous spherical particles (7 nm iron or 10 nm gold) reduce the hexane/water interfacial tension from 48 mN/m to ~33 mN/m, whereas using similar-sized Janus particles of gold and iron lowers the interfacial tension further to ~22.5 mN/m [85]. Combining shape and surface anisotropy can further enhance the adsorption of Janus particles to fluid interfaces resulting in their pronounced surface activity. For instance, Ruhland et al. synthesized Janus particles of different geometries using block terpolymers and reported that spherical Janus particles (50 nm in diameter) reduce the toluene/water interfacial tension from 36 mN/m to ~18 mN/m, whereas cylindrical Janus particles (diameter of 23 nm and length of 2300 nm) can further decrease the tension down to ~14 mN/m [47,78]. The interfacial activity of Janus particles has been used to explain their superior performance in interfacial systems, such as their role as stabilizers in emulsions and foams, schematically shown in Figure 1c [72,75,89]. As an example, Yin et al. studied the impact of Janus character for nanofluids flooding in enhanced oil recovery and showed that utilizing Janus nanosheets at an ultralow concentration of 0.005 wt.% reduces the oil/water tension and yields higher interfacial shear viscosity, which in turn can enhance the efficiency of oil recovery by more than 18% with minimal impairment to the permeability [90]. In addition to interfacial activity, Janus particles with pH responsiveness, magnetic functionality, and temperature sensitivity have opened the door to switchable interfacial systems enabling the controlled formation and breakage of emulsions on demand using external stimuli [84,91,92,93,94,95].

In many aforementioned applications, the interface also undergoes deformations that produce compression and shear stresses, as shown schematically in Figure 1b. Therefore, in addition to the physicochemical properties of the stabilizer, an effective stabilization depends on the flow behavior of the stabilizer in response to applied stresses [96]. For example, amphiphilic silica-based Janus nanoparticles (diameter of 40 nm) not only resulted in a reduction of oil/brine interfacial tension but also led to an increase of the interfacial shear viscosity such that their utilization in nanofluids (0.01 wt.%) yielded an enhancement in oil recovery by 15.74% compared to homogenous particles [97]. In stabilization of Pickering foams using Janus particles, the enhanced stability has been correlated with the high dilational elasticity and mechanical strength of the interface in presence of Janus particles [75,76]. A fundamental understanding of how particle-laden interfaces behave under flow is thus of critical importance to gain predictive control over the properties of microstructures at interfaces in order to efficiently engineer them for targeted applications. In line with this objective, interfacial rheology can be used to probe the behavior of particle-stabilized systems focusing on the attributes of particles and their impact on rheological properties of interfaces [98].

The field of rheology examines the deformation and flow of matter in response to applied disturbances. Interfacial rheology pertains to techniques investigating the behavior of interfaces (2D) to inform us on the role of surface-active species in the resulting properties of interfacial systems [99,100,101]. For instance, in the dilational rheology realm, particle-laden interfaces have shown a dominant elastic behavior in presence of fumed silica particles [102]. Upon compression of the interface, monolayers of colloidal homogenous particles have shown different collapse mechanisms depending on the particle wettability [103]. Regarding Janus particles, more complex interparticle interactions arise due to particle anisotropy, which in turn affect the resulting microstructure and its interfacial rheology [104]. The scope of this review paper is to highlight the key factors that influence the interfacial stability and dilational/shear interfacial rheology of particle-stabilized systems. We focus on studies carried out on interfacial systems composed of Janus particles. In the stability section, we provide an overview of the main factors that influence the binding energy of a particle to a fluid/fluid interface, interparticle interactions, and interfacial activity. Next, we briefly review the techniques used to probe the interfacial rheology and survey the findings reported in the literature. The factors that govern the response of an interfacial particle network to applied stresses are discussed and parallels are made between homogenous and Janus particles. In the dilational rheology section, we will discuss how interfacial systems composed of particles respond to a change in area, either compression or expansion. In the shear section, we discuss the key parameters impacting the shear behavior of particle-laden interfaces and review how fluid interfaces decorated with Janus particles respond to and are affected by interfacial shear stresses.

## 2. Stability of Particles at Fluid Interfaces

### 2.1. Equilibrium Position of Particles at Fluid Interfaces

The equilibrium contact angle $\left(\theta_{E}\right)$ of a particle at a fluid interface can be calculated by the minimization of the free energy of the system [4,106]. For homogeneous particles, this results in the well- known Young’s equation as follows:

(1) $$\cos\theta_{E}\;=\frac{\gamma_{po}\;-\;\gamma_{pw}}{\gamma_{ow}}$$

The factors that determine the interfacial positioning of the particle are thus the fluid/fluid interfacial tension $\left(\gamma_{ow}\right)$ and the surface tension of the particle with both fluid phases $(\gamma_{po}$ and $\gamma_{pw})$ [82,107,108]. Therefore, from a thermodynamic standpoint, particles spontaneously adsorb on a fluid/fluid interface, provided that the surface energy between the two fluids is greater than the difference between the surface energies of the particle with each fluid phase [109]. However, research carried out by Manoharan et al. and others on dynamics of binding has shown that adsorption of particles to a fluid interface can be characterized by a sudden breach, driven by capillary forces, followed by a slow relaxation to equilibrium that appears logarithmic in time [110,111]. It is proposed that complete equilibration of a particle to the predicted Young’s contact angle may take time due to the presence of nanoscale heterogeneities on the particle surface [112,113]. Once the particles are trapped at a fluid interface, their desorption back into the bulk requires an energy input that goes as $\Delta E=\pi R^{2}\gamma_{ow}{\left(1\pm \cos\theta_{E}\right)}^{2}$, where R is the radius of the particle. The value of desorption energy is much larger than the thermal energy, e.g., $\sim 10^{7}\;k_{B}T$ (where $k_{B}$ is the Boltzmann constant) for a 1 μm neutrally wetting ($\theta_{E}=90^{{}^{\circ}}$) particle trapped at the air/water interface; thus, in contrast to surfactant molecules, the adsorption of colloidal particles to interfaces can be considered as irreversible. For non-spherical particles, equating the volume to a spherical shape can be used for the calculation of the detachment energy as shown by Anjali and Basavaraj [114].

**Figure 2:**
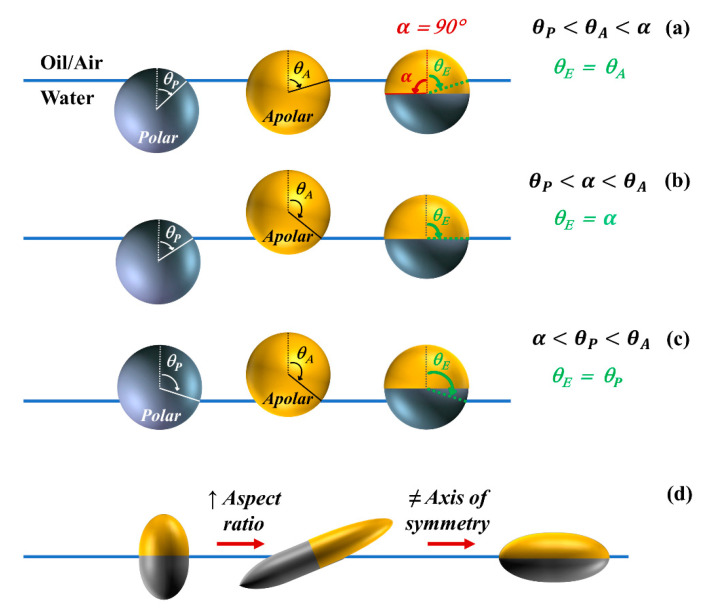
(Color online) Equilibrium configurations of a Janus particle residing at a fluid/fluid interface: the equilibrium contact angle (**a**) equaling the apolar contact angle (Equation (2)), (**b**) coinciding with the Janus boundary (Equation (3)), (**c**) equaling the polar contact angle (Equation (4)); (**d**) the impact of combined shape and surface anisotropy.

Similar to the derivation of Young’s contact angle, the equilibrium position of a Janus particle at a fluid interface can be predicted from the minimization of the free energy of the system with respect to the immersion angle [115]. Because Janus particles carry a dual chemistry—a polar face with a contact angle $\theta_{P}$ and an apolar compartment with a contact angle $\theta_{A}$—they possess an amphiphilic nature [116,117]. The degree of amphiphilicity, Δθ, for a Janus particle is defined as $\Delta \theta =\left(\theta_{A}-\theta_{P}\right)/2$. The surface boundary partitioning the polar and apolar faces is indicated by the angle α; values of $\alpha =0^{{}^{\circ}}\;$or $\alpha =180^{{}^{\circ}}$ correspond to a homogenous particle, whereas $\alpha =90^{{}^{\circ}}$ refers to a Janus particle with two equal-sized patches of different wettability, as depicted in Figure 2a [107].

Altering the particle amphiphilicity, through the wettability of each face ($\theta_{P}$ and $\theta_{A}$), or the location of the Janus boundary, through the value of angle α, will impact the particle configuration at the interface. By assuming that the Janus boundary will align parallel to the plane of the interface, i.e., disregarding the rotational behavior of the particle, the minimization of the free energy yields three possibilities for the equilibrium contact angle $\left(\theta_{E}\right)$ of a Janus particle at the interface, schematically shown in Figure 2a–c, as follows [115]:

(2) $$\theta_{P}\;<\;\theta_{A}\;<\;\alpha ,\theta_{E}\;=\;\theta_{A}$$

(3) $$\theta_{P}\;<\;\alpha \;<\;\theta_{A},\;\;\theta_{E}\;=\;\alpha$$

(4) $$\alpha \;<\;\theta_{P}\;<\;\theta_{A},\;\;\theta_{E}\;=\;\theta_{P}$$

Therefore, the wettabilities of the two faces determine the equilibrium contact angle of the particle straddling the interface ($\theta_{E}$), which in turn impacts the magnitude of the particle detachment energy from the interface (ΔE). Similar to homogenous particles, an increase in size of a Janus particle leads to a larger energy of detachment [86]. In contrast with homogeneous particles that possess an isotropic surface chemistry, the detachment energy of a Janus particle can be further enhanced by increasing the particle’s degree of amphiphilicity (Δθ), as shown by Binks and coworkers [82]. Analytical calculations illustrated that by switching from a homogeneous particle of neutral wettability (R = 10 nm, $\Delta \theta \;=\;0^{{}^{\circ}}$, $\theta_{E}\;=\;90^{{}^{\circ}}$) to a highly amphiphilic Janus particle ($\Delta \theta =90^{{}^{\circ}}$), the desorption energy is increased by approximately three-fold [82]. Inspired by the potential of Janus particles at fluid interfaces and the tunability of their behavior, research has boomed in this area with applications spanning from enhanced oil recovery to bi-phasic catalytic reactions [6,89,90,118,119]. However, the rotational behavior of Janus particles cannot be neglected as the particle stability is also influenced by its orientation at the interface [78,107,120,121,122,123,124]. Monte Carlo simulations performed by Bon and Cheung illustrated that neglecting the Janus nanoparticle rotation at the interface significantly overestimates the detachment energy and thus the orientational freedom of the particle must be considered [125]. Further studies done by Lee et al. highlighted the impact of the amphiphilicity on the Janus particle orientation at the interface [114,120]. Gold coated polystyrene Janus particles assumed random orientations at the interface, due to their low amphiphilicity, yielding small energy differences between the cap-up and sideways orientations. In contrast, for thiol-modified Janus particles, a greater energy difference exists between the cap-up and sideways orientations leading to over 90% of particles residing in a configuration where the Janus boundary was aligned with the interface.

Another factor impacting the configuration of particles at fluid interfaces is the introduction of shape anisotropy in a Janus particle. Shape anisotropic particles of uniform surface wetting will reside at the interface with equilibrium configurations that maximize the displaced area of the fluid interface; e.g., an ellipsoidal particle will lay flat on the interface with its major axis aligned with the plane of the interface [114,126]. When surface chemical anisotropy and shape anisotropy are combined in a single particle, the equilibrium configuration can be more complex as the former type of anisotropy favors maximizing the contact areas of polar/apolar regions on the Janus particle surface with the respective polar/apolar fluids, whereas the latter form of anisotropy favors a configuration in which the interface is intercepted by the largest cross-section of the solid particle [127,128]. For example, an ellipsoidal particle with a Janus boundary located parallel to the short axis of the particle will sit upright at the interface if the particle aspect ratio is low and degree of amphiphilicity is high. However, as the aspect ratio is increased, the interplay between the chemical anisotropy and shape anisotropy in minimizing the free energy will lead to a particle tilt at the interface, as illustrated in Figure 2d [78]. To take the rotational freedom of the particle into account, an analytical expression was derived by Stocco et al. for the free energy as a function of orientation of a single Janus particle assuming a flat interface [129]. Günther et al. [130] examined the impact of fluid interface deformation on the equilibrium orientation of a Janus ellipsoidal particle by comparing the theoretical predictions of free energy models to results of Lattice Boltzmann simulations. It was shown that factors such as the deformation of the interface and the adsorption process can affect the equilibrium orientation of shape anisotropic Janus particles [130]. In a more realistic scenario, the interfacially-trapped particles will also interact with each other and these interparticle interactions thus impact the particle configuration, the resulting microstructure, and the collective behavior of interfacial systems as discussed in the next section.

### 2.2. Interparticle Interactions at Fluid Interfaces

**Figure 3:**
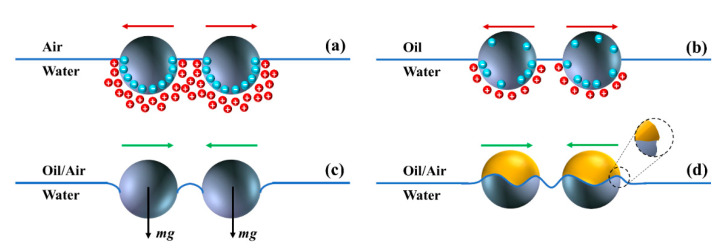
(Color online) Examples of interparticle interactions that can be present at fluid interfaces. (**a**) Dissociation of surface groups causing repulsive dipole-dipole interactions; (**b**) water entrapment on the particle surface in contact with the oil phase causing long-ranged repulsive interactions; (**c**) gravitational forces deforming the interface generating attractive capillary interactions; (**d**) interface undulations caused by surface roughness along the Janus boundary resulting in capillary interactions.

The stability of colloidal particles at fluid interfaces and the microstructure of the resulting interfacial layer is dictated by the type and relative strength of the interparticle interactions that exist in presence of an interface [131]. While the properties of adsorbed particles and fluids making up the interface are the factors determining the interparticle interactions, and consequently the stability of the monolayer, the nature and spectrum of these interactions is an active area of research. Different terms of interparticle interactions considered in the literature can be broadly classified into attractive and repulsive categories [12], examples of which are illustrated in Figure 3.

The repulsive interaction between charged colloids trapped at a fluid/fluid interface has been attributed to the dissociation of surface charges in the polar medium, which yields an asymmetric screening cloud with respect to the interface plane, shown in Figure 3a [132,133,134]. As suggested by Pieranski [108] and validated by experiments [135,136,137], the effective dipole generated by the particle surface charge and the resulting screening cloud in the polar medium leads to long-range dipole-dipole interaction between particles that scales as $F\sim \;r^{-4}$ for particle separation distance of r [132,138,139]. It has also been suggested that monopoles, originated from the dissociated surface groups in the polar phase and exposed to the non-polar phase, could contribute to the repulsive interparticle interactions as illustrated in Figure 3b [137,140,141,142].

The attractive dispersion van der Waals (VDW) interactions that originate from the fluctuations of the electron cloud around the atomic nucleus, can be calculated for colloidal particles suspended in a fluid (3D) using the value of the Hamaker constant ($A_{131}$) for particles interacting through a medium [143]. A similar formalism has been proposed for the calculation of VDW interactions between interfacially-trapped colloidal particles using the Derjaguin approximation, when the range of interactions is small compared to the particles radius of curvature [144]. It has been assumed that the VDW interactions occur between the immersed parts and the emergent parts of the particle through different fluid media, with different Hamaker constants assigned for interactions through each fluid phase.

The double layer repulsion and the VDW attraction make up the Derjaugin–Landau–Verwey–Overbeek (DLVO) potential, which dominates most aqueous dispersions and accounts for the stability of colloidal particles in bulk (3D). However, the behavior of colloidal particles at an interface (2D) is not fully captured by the DLVO approximation [145]. The retarded VDW forces are short-ranged and typically extend over a range of tens of nanometers for micrometer-sized colloids [143], while an unexpected long-ranged attraction has been reported for interfacially-trapped particles [146,147,148,149]. This strong long-ranged attractive interaction has been attributed to capillary forces [139,150,151,152,153,154,155,156,157,158,159,160] resulting from the distortions imposed on the interface by the particles, represented in Figure 3c [120,161]; an interfacial phenomena with no analogy in bulk aggregation. The interfacial distortions can originate from the weight of particles (gravity-driven capillary attraction for heavier or bigger particles) [162] or electrostatic stresses caused by the particles dipolar field (electrodipping) [158,163]. In addition, surface roughness, chemical inhomogeneity, and shape anisotropy of particles cause the meniscus to take an irregular shape over the particle surface in order to satisfy the correct contact angle at all points along the perimeter, as shown in Figure 3d [152,164,165,166,167,168]. The attractive force between two particles at a separation distance of r is shown to scale as $F\sim \;r^{-5}$, which is especially consequential in case of heterogeneous particles [139,167,168,169,170].

The relative strength of electrostatic repulsions and capillary attractions dictate the assembly behavior of colloidal particles and the resulting microstructure. Hence, by tuning the interactions and switching from a repulsive to attractive potential, clustering and aggregate formation can be stimulated [120,171,172,173]. A plethora of opportunities exists for tuning the colloidal interactions for instance through particle wettability, introducing anisotropy, addition of electrolytes, solution pH, and synergism in presence of surfactants [9,29,174,175]. For example, Horozov et al. observed that silica particles of low hydrophobicity ($\theta_{E}=65^{{}^{\circ}}$ measured through the water phase) form disordered unstable aggregates at the octane/water interface, whereas very hydrophobic particles ($\theta_{E}=152^{{}^{\circ}}$) result in a highly ordered monolayer. These results were explained in terms of a pair potential composed of contributions from electrostatic repulsion (through both polar and non-polar media) and capillary attraction (due to three-phase contact line undulations) and were attributed to the change in the magnitude of the surface charge density on the particle/octane interface as particle hydrophobicity is increased [141]. Achieving microstructures with a percolated network can then be employed in designing interfaces with a desirable stability [176]. For instance, interfacially-trapped colloidal monolayers of sufficient yield stress are shown to impact gas dissolution from the particle-coated bubbles arresting Ostwald ripening in foams [24]. Similarly, the elastic modulus of a jammed network of colloidal particles at a droplet surface is shown to offset the Laplace stress driving the fusion of droplets resulting in arrested coalescence in emulsions [177]. Recent work on Janus particles as interfacial stabilizers also reports on correlations between dilational viscoelasticity of particle-laden interfaces and foam drainage-half time, where Janus particles exhibited higher elastic modulus and outperformed systems stabilized with homogeneous particles, surfactants, or a foaming agent [75,76]. As can be seen, rheology plays a key role in performance of interfacial systems; therefore, to unlock the tremendous potential of colloidal particles at interfaces, it is important to gain a fundamental understanding on the link between interparticle interactions, especially in case of heterogeneous particles, and the interfacial rheology of the ensuing microstructure as reviewed in the next section.

## 3. Interfacial Rheology

### 3.1. Tools and Techniques

**Figure 4:**
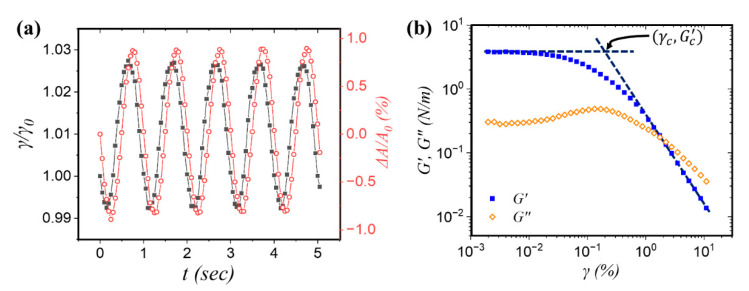
(Color online) Examples of data acquired from interfacial rheology measurements: (**a**) a bubble suspended in a 2 mM sodium dodecyl sulfate (SDS) surfactant solution and oscillated at 1 Hz. Data depicts the change in surface tension (γ), normalized by the initial surface tension value ($\gamma_{0}$), (solid black symbol) vs. time due to the applied strain (open red symbol)—variations in the surface area (ΔA) normalized by the initial area ($A_{0}$). The phase shift (δ) between the two curves is used to calculate the contributions to the complex modulus (E) as defined in Equation (5); (**b**) data on oscillatory shear rheology measurement performed using a TA Instruments Discovery Hybrid Rheometer-2 (DHR-2) via the DWR geometry (inner cup diameter: 62 mm, inner ring diameter: 69 mm, outer ring diameter: 71 mm, outer cup diameter: 79 mm, ring thickness: 1 mm). Measurements were carried out at 1 Hz on the air/water interface decorated with silica/gold Janus particles. For this dataset, 5 mg of Janus particles were suspended in 200 μL of 70/30 wt% isopropyl alcohol/water mixture that was used as a spreading solvent. After deposition, the interface was left undisturbed for 20 min to allow for the IPA evaporation. The silica/gold Janus particles were fabricated from 1 μm spherical silica particles (Fiber Optic Center, Inc.) half-coated with a 5 nm-thick adhesive layer of titanium followed by a 10 nm gold deposition. The gold face is then modified with dodecanethiol molecules to boost the amphiphilicity of the Janus particle ($\Delta \theta \;\sim 40^{{}^{\circ}}$). The solid symbol illustrates the elastic ($G^{\prime }$) contribution and the open symbol shows the viscous ($G^{\prime \prime }$) contribution vs. strain (γ) in a log-log plot. The critical strain ($\gamma_{c}$) and the low-strain plateau elastic modulus ($G_{c}^{\prime }$) were obtained using a procedure detailed in [98] and the yield stress ($\tau_{y}$) value, calculated using Equation (6), was ~0.007 Pa.m for this sample [186].

Interfacial rheology studies the flow behavior of fluid interfaces in order to investigate the response of adsorbed species (e.g., particles, surfactants, polymers, proteins, etc.) subjected to an applied deformation, in form of changing either the area (i.e., dilational rheology) or shape (i.e., shear rheology) of the interface [178,179,180,181]. Similar to bulk rheology, the interfacial rheological techniques can be categorized into strain-controlled and stress-controlled instruments [182]. Strain-controlled instruments operate by applying a prescribed strain γ (or strain-rate $\dot{\gamma }$) to the interface and measuring the stress response, σ. The applied strain can be constant in time or changing as a function of time. For example, in step-strain measurements used to probe the stress relaxation in the material, a known rapid strain is applied followed by the measurement of the stress response as a function of time. In oscillatory measurements, the interface is subjected to sinusoidal strain of a given frequency and the interfacial stress is monitored [183,184,185]. The phase shift (δ) between the applied sinusoidal strain and the measured stress (in a strain-controlled rheometer), illustrated in Figure 4a, can be used to determine the elastic and viscous moduli. Depending on the material, the response may be purely elastic (stress is proportional to the strain, phase angle of $\delta =0^{{}^{\circ}}$), purely viscous (stress is proportional to the strain rate, $\delta =90^{{}^{\circ}}$), or viscoelastic ($0^{{}^{\circ}}<\delta <90^{{}^{\circ}}$). The storage modulus provides information on the presence of structure in the sample and describes the energy stored in such structure, whereas the loss modulus characterizes the energy dissipated in the sample and represents the viscous nature of the material.

In dilational interfacial rheology, the area perturbations can be carried out by either a continuous surface compression at a specified constant rate or oscillatory compression/expansion of the interface. The interfacial area (A) is altered and the resulting change in the surface stress or surface tension (γ) is measured (as shown in Figure 4a) and captured in the complex dilational modulus (see Equation (5)) [183]. The complex dilational modulus (E) can be split into elastic ($E^{\prime }$) and viscous ($E^{\prime \prime }$) contributions as follows:

(5) $$E\;=\;\frac{d\gamma }{dlnA}\;=\;E^{\prime }\;+\;iE^{\prime \prime }\;\equiv \;E^{\prime }\;+\;i\omega \eta_{d}$$

where ω is the frequency of oscillations and $\eta_{d}$ is the surface dilational viscosity. Interfacial shear rheology examines the response of interfaces to shear stresses. Similar to the dilational experiments, a complex shear modulus ($G^{*}$) can be defined as $G^{*}\;=\;G^{\prime }\;+\;iG^{\prime \prime }$, where $G^{\prime }$ and $G^{\prime \prime }$ are the storage and loss moduli; respectively, as shown in Figure 4b [187]. The yield stress, at which viscosity decreases sharply, can be measured and verified by strain amplitude sweep, stress ramp, and creep experiments [98]. In amplitude sweep measurements, as illustrated in Figure 4b, the critical strain ($\gamma_{c}$) beyond which the interface enters the nonlinear viscoelastic regime is determined by the intersection of the low-strain plateau elastic modulus ($G_{c}^{\prime }$) and a power law fit to the data at high strain. From this information, the yield stress ($\tau_{y}$) can be estimated as:

(6) $$\tau_{y}\;=\;\gamma_{c}G_{c}^{\prime }$$

The challenge in designing an interfacial rheometer is the coupling between the flow profile at the interface and that in the surrounding bulk phases [188]. Interfacial rheology experiments are therefore considered more difficult than bulk rheology [99]. To minimize the influence of the bulk phase on the measurements and resulting data, interfacial rheometers rely on the design of geometries that reduce the sub-phase drag contribution relative to that of the surface drag as captured in Boussinesq number (*Bo*) defined as follows:

(7) $$Bo\;=\;\frac{surface\;drag}{subphase\;drag}\;=\;\;\frac{\eta_{s}}{\eta *a}$$

in which $\eta_{s}$ is the surface shear viscosity, η is the sub-phase bulk viscosity, and a is a characteristic length scale calculated from the dimensions of the geometry [188]. It should be noted that the Boussinesq number, defined earlier for the interfacial shear rheology, has an analogue for the interfacial dilational stresses relative to the bulk stresses [189,190]. When Bo≫1, the interfacial stresses dominate, and the surface rheology is captured. This can be accomplished by minimizing the value of a through a geometry design that maximizes the perimeter of contact between the probe and the interface for a given contact area of the probe with the sub-phase [188].

**Figure 5:**
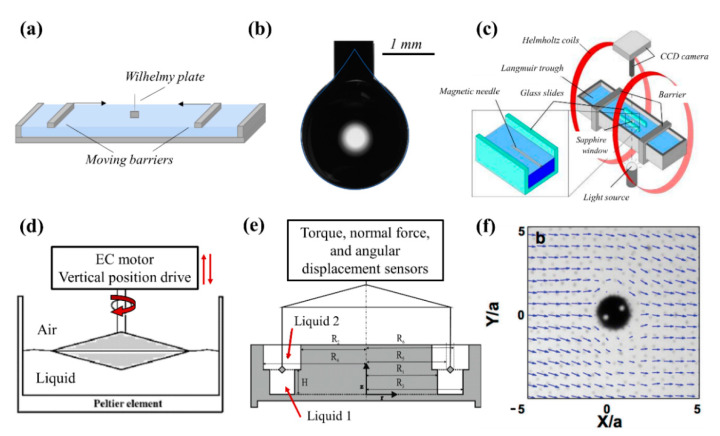
(Color online) Examples of techniques used to perform interfacial rheological measurements. (**a**) Langmuir Trough—measures the pressure at the interface in response to applied compression and expansion; (**b**) Pendant Drop—fits the shape of a droplet hanging from a needle in response to expansion and contraction of its volume; (**c**) Magnetic Needle—measurements of interfacial rheology by shearing the interface with a needle-like probe using an externally applied magnetic field; (**d**) Bicone—shearing of the interface with a conical shaped probe; (**e**) Double-walled Ring geometry (R_1_-R_4_ define the dimensions of the cup, R_5_ and R_6_ indicate the ring dimensions, and H is the distance from the interface to the bottom of the circular channel) —shearing of the interface with a ring; (**f**) Microrheology—indirect measurements of rheological properties utilizing a microparticle probe pinned at the fluid interface. The radius of the micro-probe, a, is 50 μm in this figure. Panel (**c**) reprinted with permission from [202], copyright 2021 American Chemical Society. Panel (**d**) reprinted from the open access [184]. Panel (**e**) reprinted by permission from [Springer Nature]: [MDPI AG] [Rheologica Acta] [204] [Copyright 2021]. Panel (**f**) reprinted with permission from [206], copyright 2021, The Society of Rheology.

To measure the response of interfaces to changes in the area, a number of techniques including Langmuir trough (Figure 5a), pendant drop tensiometer (Figure 5b), and the capillary wave technique can be used [178,191]. Langmuir balance is a versatile technique that can be employed to measure the interfacial activity of particles [191], examine their microstructure at fluid interfaces [192,193], and probe the mechanical response of interfaces subjected to 1D compressions and expansions [3,194]. This method measures the surface pressure (i.e., the difference between the interfacial tension in presence of surface-active species and that of bare fluid interface) using a Wilhelmy plate attached to a balance and monitors the change in the surface pressure (Π) as the interfacial area (A) is altered. The resulting information is recorded as pressure vs. area isotherms. Analogous to 3D systems, the static compression modulus of the interface ($E_{0}$) can be calculated by taking the derivative of surface pressure with respect to interfacial area at a constant temperature [3] as follows:

(8) $$E_{0}\;=\;-A{\left(\frac{\partial \Pi }{\partial A}\right)}_{T}\;=\;-{\left(\frac{\partial \Pi }{\partial lnA}\right)}_{T}$$

Pendant drop tensiometry relies on a geometrical fit of the drop shape to the Young-Laplace equation, which balances gravitational forces with surface forces. This instrument is widely used to monitor the interfacial tension as a function of time, which can yield insight on the adsorption and desorption processes of surface-active species onto the interface [87,195,196,197], but can also be adapted to probe the interfacial rheology [181,198]. The drop (or bubble) is subjected to volume changes, which consequently results in changes of the surface area. The advantage of this technique is that less particles are needed to cover the interface (A ~tens of mm^2^ for droplets of ~10 μL) compared to a Langmuir trough (A ~hundreds of cm^2^ with volumes ~500 mL) [199]. Moreover, instead of uniaxial compression/expansions, the pendant drop technique allows for a more uniform change of the surface area. The capillary wave technique has also been used for dilational interfacial rheology and is discussed in more details elsewhere [179,191]. For shear interfacial rheology, magnetic needle (Figure 5c) [200,201,202], interfacial disk/bicone (Figure 5d) [203], and double-wall-ring (DWR) (Figure 5e) [204] geometries can be used to probe the properties of the interface in response to applied shear deformations [205]. Microrheology is also utilized in sensitive surface shear rheology measurements, where a ferromagnetic micro-probe pinned to a fluid/fluid interface is actively torqued or forced using externally controlled electromagnets (Figure 5f) [206,207]. A more detailed discussion on each technique and their limitations can be found elsewhere [178,208].

Interfacial rheology experiments can be conducted either with particles dispersed in the bulk phase and diffusing to the interface forming a so-called Gibbs monolayer, or deposited directly at the interface, generating a Langmuir monolayer [209]. Factors that need to be considered with the former method are the time required for particles to diffuse to the interface, energetic barriers to adsorption, and the relaxation of the adsorbed particles into their equilibrium configuration [111,210]. In a diffusion-driven process, it takes ~1 s for a 1 μm colloidal particle suspended in water to diffuse its own radius, whereas for a 10 μm particle the required time increases to $10^{3}\;$s. In addition, the stability of particles to sedimentation also needs to be considered. Peclet number (Pe) —the ratio of convective to diffusive transport—is ~0.1 for a 1 μm silica particle suspended in water, whereas for a 10 μm particle,$\;Pe\;\sim \;10^{3}$. The diffusion process is followed by the adsorption step, which can be hindered by a repulsion between charges on the particle surface and the interface [111]. There is evidence that even when the charge interaction between the particle and the interface is attractive, adsorption can be hindered by the electrostatic force resulting from an image charge [12,210]. The image charge effect is not always repulsive, as it has been observed to cause either repulsive or attractive interactions in particle-interface systems depending on the dielectric constant of medium the particle is initially suspended in compared to that of the neighboring phase [12,211]. Finally, after breaching the interface particles experience a relaxation process towards the equilibrium contact angle, $\theta_{E}$, which can take up to months, suggesting that experimental time frames may not be capturing the equilibrium state of particles at the interface [111]. The interfacial deposition method relies on the use of a spreading solvent to disperse the colloidal particles at the interface via Marangoni flow. However, the choice of spreading solvent is shown to impact the contact angle of particles at the interface, thus, different results may be obtained based on the solvent used for this technique [212,213]. Fernandez et al. [86] studied the role of spreading agent on the interfacial entrapment of particles and concluded that any amount of water in the spreading solvent was not beneficial as the particles would fall through the interface during the deposition process resulting in a reduced entrapment efficacy. The trapping of particles at the interface is therefore an important parameter to consider. It is a common procedure to assume that all particles suspended in the spreading solution will be trapped at the interface upon deposition. This assumption is used to estimate either the area available per particle or the surface concentration of particles in the system under study. However, care must be taken to consider the role of solvent and the particle surface properties on the entrapment efficacy of particles. This is critical when comparing the interfacial behavior for particles of different characteristics. For instance, hydrophilic colloidal silica particles, which are negatively charged at neutral pH due to dissociation of surface silanol groups, are more likely to fall through the air/water interface upon deposition at the interface given that the interface carries negative charges [214]; the presence of electrolyte in the sub-phase is shown to enhance the entrapment efficacy of particles in this case and the mechanical properties of the resulting interfacial layer [103,215]. Variation in binding efficacy has also been reported for Janus particles with different degrees of amphiphilicity [107].

### 3.2. Interfacial Dilational Rheology

#### 3.2.1. Homogeneous Particles

The dilational rheology of homogeneous particles at fluid interfaces is affected by parameters such as wettability, surface coverage, and shape anisotropy that dictate the microstructure of the interfacial layer [36,98,216]. Studies on the impact of particle wettability and interparticle interactions on the resulting dilational behavior have been carried out on various particle types including gold, polystyrene, and silica particles [102,197,216,217]. For example, silica particles are hydrophilic due to their surface silanol (SiOH) groups ($\theta_{E}\;\sim 20^{{}^{\circ}}$ for 100% SiOH) but their wettability can be altered by replacing the silanol groups with grafted alkyl chains via silanization process ($\theta_{E}\;\sim 110^{{}^{\circ}}$ for 20% SiOH) [218]. Using a Langmuir trough, it was shown that the monolayer of hydrophilic silica particles (20 nm and $\theta_{E}\;\sim 40^{{}^{\circ}}$) at the air/water interface exhibited a 2D compression modulus of ~40 mN/m, whereas by increasing the hydrophobicity of particles ($\theta_{E}\;\sim 90^{{}^{\circ}}$) the modulus increased to ~100 mN/m [216]. The reported surface pressure isotherms suggested a superior trapping efficiency for more hydrophobic particles. The repulsive interaction between the negatively charged hydrophilic particles and the interface contributed to their poor binding to the interface. In addition, the hydrophobic particles require a larger energy input to desorb from the interface compared to hydrophilic particles. The same trend is observed by Safouane et al. [102] for monolayers of fumed silica particles (200 nm sized aggregates) using capillary waves at high frequencies (400 Hz); while the static compressibility modulus of hydrophilic particles did not exceed 2.5 mN/m, data for hydrophobic particles exhibited a maximum of ~186 mN/m. Razavi et al. [103] studied larger particles (1 μm) at the air/water interface under compression using a Langmuir trough and investigated the effect of particle wettability and the presence of electrolyte in the sub-phase on the collapse mechanism. Collapse of interfacial layers was obtained by a sustained compression of the interface to areas smaller than that corresponding to a close-packed 2D network. It was highlighted that the hydrophobic monolayers formed a solid-like network that collapsed via buckling, whereas the hydrophilic particles resulted in a fluid-like monolayer due to strong repulsive interparticle interactions that collapsed via particle expulsion to the sub-phase. The screening of electrostatic interactions, present in the latter case, was achieved by the addition of electrolyte to the sub-phase, which led to the collapse of the network via multilayer formation at the interface. The compressional modulus of the monolayer, obtained from the differentiation of the pressure isotherms, was initially lower for layer composed of hydrophobic particles compared to that of hydrophilic particles. However, after successive compression-expansion cycles, the modulus of the hydrophilic layer declined due to the sustained particle expulsion from the interface to the bulk, whereas the hydrophobic monolayer exhibited an opposite trend, due to particle clustering and network formation. The depletion interaction provided by dissolving a hydrophilic polymer (e.g., poly(ethylene glycol) (PEG)) in the sub-phase is also shown to improve the binding of hydrophilic colloidal silica particles to the air/water interface and yield a collapse mode in form of multilayer formation [219]. The interested reader can seek information on extreme deformation of particle-laden interfaces and the resulting collapse mechanisms in the review paper by V. Garbin [220].

While particle wettability can be altered by the chemical modification of the surface (e.g., silanization in case of silica particles), an alternate mechanism for tuning the particle wettability is through the adsorption of surfactants onto the solid particle surface [221]. Air/water interfaces with mixed particle and surfactant systems (silica particles and the cationic surfactant Cetyl Trimethyl Ammonium Bromide (CTAB)) exhibited an enhanced rigidity after 24 h of aging without any alterations observed for the air/water surface tension. The modulus of dilational elasticity increased from ~40 mN/m to ~1000 mN/m when the CTAB concentration was increased from 0.02 mM to 0.5 mM. The authors discuss that multilayers of particles are forming at the interface due to the surfactant adsorption onto the particle surface, which causes a decrease in the particle surface charge [221]. In addition, the surfactant adsorption on the surface of particles renders them more hydrophobic, which is responsible for the irreversible attachment of particles to the air/water interface, resulting in an interfacial layer that behaves in a solid-like manner [222].

The impact of particle wettability on the dilational modulus of interfaces has been used to control the performance of interfacial systems. For instance, the presence of partially hydrophobic fumed silica particles (diameter of 14 nm) in foams made with anionic surfactants (a commercial surfactant Hengye-2) was reported to increase the oil recovery for heavy oil production from ~43% (in the absence of particles) to ~68% (with 1 wt.% of particles). The improved performance was attributed to the increase in interfacial dilational viscoelasticity with the addition of the silica nanoparticles (a factor of ~6 for 1 wt.% particles) [223].

The particle size and size distribution can also influence the properties of particle-laden interfaces and their response to compression. The Young modulus for a monodispersed close-packed monolayer of particles, derived by Vella et al., is inversely proportional to the particle diameter [224]. Using maghemite particles (average size of 7.5, 11 and 15.5 nm) Lefebure et al. [225] showed that small particles are more compressible than larger particles. This is in agreement with the study carried out by Bykov et al. [226] on polystyrene particles (100 nm and 1 μm diameter) at octane/water interfaces with different ionic strengths of the sub-phase. It was found that the dynamic surface elasticity of the monolayers is slightly dependent on the size of the particles due to the more compact packing of smaller (100 nm) particles. The presence of polydispersity in particle-laden interfaces also impacts how monolayers react to compression, as shown by Yang et al. [227]. Soda-lime glass particles (diameter of 150 μm, 64 μm, and 96 μm) were used to make five different samples with different size ratios at the air/water interface. It was found that the surface pressure isotherm of unimodal samples is slightly steeper and shifted to a lower normalized area when compared to bimodal samples indicating a more uniform structure for the unimodal samples. It was concluded that the compressibility is independent of the degree of disorder in the layer; however, it was found to be dependent on the surface coverage.

For a given surface wettability, the surface coverage plays an important role on the rheological response of interfaces as shown by Miller et al. [228], where equation of states and dilational elasticity models were derived for both particle and particle-surfactant laden interfaces depending on surface coverages, relating the dilational rheology to the adsorption isotherms. Pawar et al. [177] illustrated that the number of particles pinned at the interface, i.e., the surface coverage, is critical for emulsion stability. Three possibilities were proposed for coalescence in experiments of bringing two similar-sized oil droplets (ranging from 50–200 μm) covered with silica particles (diameter of 1.5 μm) into contact. Considering that each droplet has a surface coverage of particles ($\Gamma_{1},\;\Gamma_{2}$) ranging from 0% to 100% (or $0\;\leq \;\Gamma_{i}\;\leq \;1$), it was shown that a total coalescence would take place if the sum of the surface coverages is lower than 1.43 ($\Gamma_{1}\;+\;\Gamma_{2}\;<\;1.43$). If $1.43\;<\;\Gamma_{1}\;+\;\Gamma_{2}\;<\;1.81$, the two droplets will coalesce until the interface is jammed at which point the coalescence is arrested and the deformed surface is sustained by the particles in the jammed state. Finally, when the sum of the surface coverages is higher than 1.81 ($\Gamma_{1}\;+\;\Gamma_{2}\;>\;1.81$), there is a total stability in the emulsion and the droplets cannot coalesce due to the jammed state present on each droplet surface.

The effect of surface coverage has also been examined for shape anisotropic particles. Beltramo et al. [98] studied ellipsoidal polystyrene particles at the air/water interface to investigate the impact of surface coverage on the compressional modulus and highlight the role of shape anisotropy. The interfacial layer of ellipsoidal polystyrene particles (major axis 2.48 ± 0.15 µm, minor axis 0.45 ± 0.03 µm) exhibited a steady increase in compressional modulus with surface coverage (with highest $E_{0}\;\sim$ 80 mN/m at 90% coverage). In comparison, spherical particles (diameter 0.82 µm) required a higher surface coverage (ϕ ~0.5) to form a percolated network; therefore, ellipsoidal particles exhibited a higher elastic modulus (~30 mN/m) compared to spherical particles at intermediate surface coverages (0.4–0.6). This behavior was attributed to the early formation of a network by the shape anisotropic particles. However, at higher coverages (ϕ >0.7), the interfacial network of spheres yielded a much higher compressional modulus ($E_{0}\;\sim 300$ mN/m for ϕ ~0.75).

Biological and biocompatible alternatives for interface stabilization have also gained attention recently and their impact on interfacial rheology is of interest [229,230]. Bertsch an co-workers [231] studied cellulose nanocrystals (CNC) at the air/water interface and showed that the interaction between these anisotropic particles at the interface is a major factor on the viscoelastic response of the layer. Their results illustrated that at a low surface coverage and low ionic strength, which yield a repulsive interparticle interaction, $E^{\prime }$ is negligible. By increasing the surface coverage and the ionic strength, due to the screening of the repulsive interactions, a transition from a fluid-like to a soft solid-like behavior was observed as demonstrated by the Lissajous plots. There is also evidence that more hydrophobic CNC exhibit a more pronounced strain hardening process upon compression [232]. This fluid-like to solid-like transition of the monolayer behavior has also been reported for spherical silica particles at the air/water interface by tuning either the wettability of particles or the addition of electrolytes in the sub-phase [105]. These findings illustrate how the interparticle interactions can be tuned to affect the microstructure of the interfacial layer and its mechanical properties. For more details on the interfacial dilational rheology of homogeneous particles we refer the reader to appropriate literature available [3,174,183,233,234,235,236].

#### 3.2.2. Janus Particles

**Figure 6:**
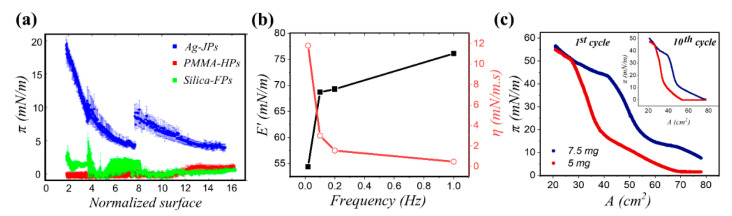
(Color online) Examples of dilational rheology response of Janus particle-laden fluid interfaces. (**a**) Surface pressure isotherms for different particles at decane/water interface. In this study, the Janus particle samples (Ag-JPs) exhibited a higher surface pressure when compared to both homogeneous particle samples; i.e., poly(methyl methacrylate) (PMMA-HPs) and silica particles functionalized with methacryloxypropyltrimethoxysilane molecules (Silica-FPs). The normalized area is defined as the total drop area divided by the area occupied by the nanoparticles assuming hexagonal packing at the interface and 100% binding efficiency upon deposition at the interface; (**b**) Reproduction of Figure 6 from [237] depicting both elastic modulus (E’) (solid black symbol) and interfacial viscosity (η) (open red symbol) for the highest concentration of particles in (**a**) deposited at the interface $(2.2\;\times \;10^{5}\;{\mathrm{nm}}^{2}/\mathrm{particle}$). (**c**) Surface pressure isotherms for high amphiphilicity Janus particles at different concentrations deposited at the air/water interface. Main figure and the inset display the first and tenth cycle of compression, respectively. Panel (**a**) reprinted with permission from [87], copyright 2021 American Chemical Society. Panel (**b**) adapted from [237] copyright 2021, with permission from Elsevier. Panel (**c**) taken from [107], with permission from the American Chemical Society.

Amphiphilicity of Janus particles is an essential attribute for their application in interfacial systems. To highlight the role of the amphiphilic character, Fernandez et al. [87] studied three systems of particles at the decane/water interface: homogeneous poly(methyl methacrylate) particles (105 nm, $\theta_{E}\;=\;76^{{}^{\circ}}$), silanized silica particles (208 nm, $\theta_{E}\;=\;94^{{}^{\circ}}$), and silver Janus particles half capped with decanoic acid (175 nm, $\theta_{E}\;=\;86^{{}^{\circ}}$). Cyclic compression/expansions of a pendant droplet of water in a medium of oil, coated with particles deposited at the interface via a spreading solvent, resulted in pressure-area isotherms displayed in Figure 6a. The Janus particle system exhibited a higher surface pressure reading at all concentrations in comparison to the two homogeneous systems. As the size of particles used in this study is similar and their contact angle values are comparable, the higher surface pressure reading observed for Janus particles was attributed to their enhanced attachment to the interface and significantly higher interfacial activity. The authors report that a 100-fold increase in the particle concentration was necessary for the homogenous particles to obtain a surface pressure isotherm similar to that of Janus particles at the oil/water interface. Several studies have reported that by depositing the same concentration of nanoparticles at the interface followed by the interfacial compression, Janus particles exhibit a higher surface pressure than their homogenous counterpart, when compared to the same area available per particle [85,87,199,237]. However, a recent study investigated the impact of synthesis and modification route on the interfacial activity of Janus particles and reported that although the Janus character seems to be a necessary condition for the reduction of interfacial tension, it is not necessarily a sufficient factor [88].

In addition to interfacial activity, the amphiphilic nature of Janus particles also impacts their configuration at fluid interfaces, which, in turn, affects the microstructure of the interfacial layer and its resulting mechanics. Kadam et al. [238] studied the dilational rheology of several different biofunctionalized silica Janus particles (diameter between 80–160 nm) at the air/MES buffer (2-(*N*-morpholino)ethanesulfonic acid) interface with a pendant drop tensiometer. It was shown that at a small strain amplitude ($dA/A_{0}\;=\;1\%$) and frequency of 1 Hz, the elastic contribution was enhanced, when compared to the untreated silica particles. Specifically, $E^{\prime }$ increased from ~2.5 to 15 mN/m, in two cases (Janus azidosilane–ferritin/biotin-PEG silane—streptavidin and Janus azidosilane–ferritin/biotin-PEG silane) in which more surface-active particles were used as demonstrated by the resulting reduction in the surface tension. In this study, all systems yielded negligible values of viscous modulus. Fernandez et al. [237] examined the behavior of poly(methyl methacrylate) (PMMA)/poly-tert-butylmethacrylate(PtBMA) Janus particles (diameter of 172 nm) at both air/water and decane/water interfaces in comparison with PMMA and PtBMA homogeneous particles. Comparing the results of surface compression from the Janus system with the homogeneous counterparts, besides having the highest surface pressure at the entire range of area, Janus particles had the higher static compressional modulus ($E_{0}$), indicating the presence of a network. With regards to surface coverage, assuming a 100% binding efficiency, it was reported that increasing the amount of added Janus particles to the air/water interface (from 5.1 × 10^5^ to 2.2 × 10^5^ nm^2^/particle) yielded an increase in both dilational viscosity (from 2 to 12 mN/m^2^) and elastic modulus (from 20 to 55 mN/m) as shown by rheology measurements carried out via 1 μL oscillations in the drop volume (with an initial volume of 45 μL) at a frequency of 0.02 Hz. In addition, the viscoelasticity of the monolayer was examined using frequency sweep measurements at a given surface coverage; the storage modulus ($E^{\prime }$) exhibited a maximum at high frequencies and reduced with decreasing the frequency of oscillations, whereas the surface viscosity displayed the opposite trend, plotted in Figure 6b.

While these studies suggest that the dilational moduli of interfacial layers are enhanced by employing a Janus character, Razavi et al. [107] further investigated the role of Janus character, specifically the impact of the particle’s degree of amphiphilicity (Δθ), in dilational properties of particle-laden interfaces. Two sets of Janus particles were studied: 1 μm silica particles capped with a 20 nm-thick layer of gold (low-amphiphilicity,$\;\Delta \theta \;\sim 20^{{}^{\circ}}$) and Janus particles with a thiolated gold cap (high-amphiphilicity, $\Delta \theta \;\sim 40^{{}^{\circ}}$). The high-amphiphilicity Janus particles exhibited a remarkable binding efficacy (>90%) upon deposition at the air/water interface using a spreading solvent in comparison to less efficient binding of the low-amphiphilicity Janus particles (30–50%). The former particle type yielded a more elastic monolayer at the air/water interface in which the cap-up orientation of particles was preserved under successive compression/expansion cycles. This layer collapsed reversibly by buckling under the applied pressure where particles remained attached to the interface with a small loss to the sub-phase when subjected to successive compression/expansion cycles (<10%), illustrated in Figure 6c. The dilational elasticity of such a Janus monolayer, which stands for its ability to store the energy applied during compression and release it upon expansion, was characterized with a compressional modulus of 167 ± 4 mN/m. In contrast, low-amphiphilicity Janus particles assumed random side-ways orientations at the interface and experienced much larger particle loss under compression (20–50%) through irreversible particle expulsion into multilayers, which underscores the critical importance of particle amphiphilicity on the rheology of the resulting interfacial layer.

Not only the Janus particle amphiphilicity can be tuned by altering the wettability of the cap but also through the core particle. As a parallel to the previous study, Lenis et al. examined interfacial monolayers of sulfonated polystyrene particles (2.4 µm) capped with a 20 nm thick gold layer and reported that the film collapsed via subduction under compression [239]. Electron microscopy images evidenced that most of the gold caps were not pointing up, and the side-ways orientation of the particles led to a random stress tensor at the interface that caused the subduction. As a means of comparison, examination of the complementary polystyrene/thiolated gold cap particle system could provide useful information to shed further light on the role of Janus balance.

### 3.3. Interfacial Shear Rheology

Both dilation and shear flows may be encountered in real applications and play a role in the stability of the interfacial systems. Van Hooghten et al. [240] studied the rheology of oil/water interfaces decorated with sterically stabilized poly(methyl methacrylate) particles and found that aggregate formation at the interface was not a necessary condition for stable quiescent Pickering emulsions in which dilational rheology dominates. However, it was reported that solid-like stiff interfaces formed by interfacial aggregates may enhance the stability of Pickering emulsions for systems in which the stability relies on the interfacial shear rheology. In this section, we provide an overview of studies carried out on interfacial shear rheology of particle-laden interfaces [215], in particular Janus systems, and their linear viscoelastic behavior. Response of interfaces containing particles to large amplitude oscillations has also been examined in the literature [241,242].

#### 3.3.1. Homogeneous Particles

**Figure 7:**
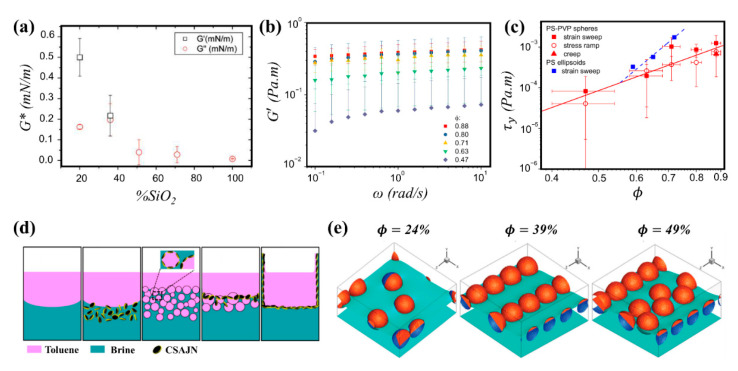
(Color online) Examples of shear rheology response for particle-laden fluid interfaces. (**a**) Impact of particle wettability, varied with altering the amount of silanol groups on the silica particle surface, on the complex interfacial shear modulus ($G^{*}$); (**b**) dependence of the elastic shear modulus ($G^{\prime }$) on frequency of oscillations and surface coverage, a higher coverage yields a more elastic behavior; (**c**) comparison between the yield stress of interfaces decorated with spheres (red symbol) and ellipsoids (blue solid symbol) as a function of the surface coverage; (**d**) formation of CSAJN Pickering emulsion in toluene and brine systems and the interfacial stability provided due to the interfacial film formation and climbing process; (**e**) shear-induced orientation and chaining of Janus particles at fluid interfaces simulated at different surface coverages. Panel (**a**) reprinted with permission from [102], copyright 2021 American Chemical Society. Panels (**b**,**c**) reprinted from [98]. Panel (**d**) reprinted from [90] with permission from Elsevier. Panel (**e**) reprinted with permission from [258], copyright 2021 by the American Physical Society.

Wettability of particles is shown to affect the viscoelasticity of particle-laden interfaces. Safouane et al. [102] studied the effect of hydrophobicity on the interfacial shear response of silica particles at the air/water interface at a constant surface concentration (Γ = 56 mg/m^2^). Probing the interfacial behavior was ensured in a surface shear rheometer using a torsion wire with a corresponding $Bo\;>\;10^{5}$. For lower to intermediate degrees of hydrophobicity ($20^{{}^{\circ}}\;<\;\theta_{E}\;<\;100^{{}^{\circ}}$), both storage and loss shear moduli were negligible. Increasing the hydrophobicity $(\theta_{E}\;=\;120^{{}^{\circ}}$) led to $G^{\prime }\approx G^{\prime \prime }\approx 0.2\;$mN/m corresponding to a gel point. With higher degree of hydrophobicity ($\theta_{E}\;=\;135^{{}^{\circ}}$), $G^{\prime }$ overcame $G^{\prime \prime }$ and the layer became stiffer due to enhanced attractive interparticle interactions. The elastic contribution was shown to be dominant for highly hydrophobic particles at the air/water interface as depicted in Figure 7a.

The particle surface coverage is also an important factor when considering the shear response of interfacial monolayers. Beltramo et al. [98] examined the behavior of polystyrene particles (~1 μm) using a Langmuir ribbon trough combined with optical microscopy and oscillatory shear rheometry. The particles were stabilized with randomly adsorbed polyvinylpyrrolidone chains to provide an uncharged steric stabilization layer and generate an increased lateral capillary interparticle interaction due to contact line undulations at the air/water interface. Their findings illustrated that increasing the particle surface coverage from 0.47 to 0.88 resulted in an enhancement of $G^{\prime }$ by one order of magnitude (from 30 mN/m to 350 mN/m), and the yield stress (from 0.1 mN/m to 1 mN/m) as shown in Figure 7b. It was stated that for particle surface coverages of Γ <0.47 no network was created, and therefore no yield stress was measured. Cicuta et al. [243] studied the behavior of polystyrene particles (~3 μm, 9.1 μC/cm^2^) at the decane/water interface with the aid of a magnetized rod rheometer (frequency of 1 Hz and amplitude of 3%) to probe the shear complex modulus ($G^{*}$). It was found that at low surface coverages, $G^{*}$ was negligible but exhibited an initial upturn at a surface coverage of 0.64, with the viscous contribution dominating. At coverages of around 0.75 to 0.80, the viscoelastic modulus reached a plateau, associated with the jamming of the particle network at the interface. These results were correlated to the bulk rheology of colloidal hard spheres, more specifically the shear-thinning behavior at high frequency limit [244]. The shear-thinning behavior has also been reported for percolated particle-laden interfaces at surface coverages below jamming. Hydrophobic sulfate polystyrene particles (diameter 3.1 ± 0.2 μm) were studied at the air/water interface ($\theta_{E}\;=\;117^{{}^{\circ}}$), where the presence of a monovalent salt NaCl in the sub-phase at 0.4 M concentration reduced repulsive interparticle interactions and allowed for the formation of dense, tightly bound particle aggregates at the interface. At low surface coverage (Γ = 0.6), the shear-thinning behavior was attributed to the breakup of initial densely-packed aggregates into smaller and disordered clusters under increasing shear rates. At higher surface coverages (Γ >0.7), some degree of shear induced ordering of particles into hexagonal packing was observed. The existence of a slip plane separating high and low shear rate zones was an indicator that the shear thinning behavior stems from yielding of the interface. The transition to yielding was reported to take place at 0.6 < Γ < 0.7, which is lower than the surface coverage required for jamming (Γ=0.77) [245]. The rheological behavior at the interface appeared to be regulated by the mesostructural organization of the microstructures highlighting the importance of local interparticle interactions in tailoring desirable rheology in interfacial systems [246]. It should be noted that using the same polystyrene particles, aggregation has also been achieved at the decane/water interface through the addition of 0.1 M NaCl and 0.1 mM SDS surfactant in the sub-phase. Study of interfacial rheology in these systems demonstrated that the polystyrene layers exhibit a dominantly elastic response, where $G^{\prime }$ has a power law dependency on the surface coverage; however, both storage and loss moduli values were smaller for particle layers at the decane/water interface compared to the air/water particle-laden interface [247].

The effect of polydispersity on the shear rheology of particle-laden interfaces is also an important parameter. Yielding transition has been reported for 2D soft glassy systems that can be characterized with nearly constant storage and loss moduli that give way to flow at high strain amplitudes [248]. For instance, Keim et al. studied disordered soft jammed interfacial structures formed by a bi-disperse mixture of 4.1 and 5.6 μm particles adsorbed at an oil/water interface with surface fraction of Γ ~0.43. Below strain amplitude of γ <0.03, the elasticity of the structure was conserved despite the presence of microstructural rearrangements, whereas above this threshold value of strain, the material was fluidized and began to lose rigidity [249].

Particles at fluid interfaces can expand their applications by adding anisotropy to the system whether through particle shape or surface chemistry [239]. Several authors have studied the effect of shape anisotropy on shear rheology of particle-laden interfaces [247,250,251,252,253]. The role of particle shape anisotropy in shear rheology has been investigated by comparing the behavior of spheres to that of ellipsoids and shape anisotropic particles are shown to be more effective in jamming at fluid interfaces [250,254]. When compared to spherical counterparts, ellipsoidal particles form a jammed network at lower surface coverages, with the threshold decreasing as non-sphericity increases [255]. In addition, ellipsoidal particles are shown to undergo buckling transition at higher surface coverages compared to spherical particles [251]. For a comparable surface coverage in both systems, network of ellipsoidal particles displayed a greater yield stress as shown in Figure 7c [98,256]. The resulting yield stress of the interfacial layer at even lower surface coverages was shown to suffice for arresting the dissolution of gas from particle-coated bubbles suspended in water, which could inhibit Ostwald ripening in foams. Further information on rheology of homogenous particle systems can be found elsewhere [180,183,257].

#### 3.3.2. Janus Particles

The link between the Janus character of particles and the shear response of the interfacial layer is an active area of research in the field to better understand and engineer the performance of resulting interfacial systems. For instance, Yin et al. examined the impact of the Janus character in nanofluid flooding for enhanced oil recovery [90]. The carboxyl/alkyl silica-based amphiphilic Janus nanosheets (CSAJN) were synthesized by a surface sol-gel process of the self-assembled monolayer of an amphiphilic silane onto a calcium carbonate (CaCO_3_) template particle that resulted in an ultrathin flake-like microstructure (~0.6 μm long and 2.6 nm thick). To probe the role of CSAJN amphiphilicity, the behavior was compared to that of non-amphiphilic silica-based Janus nanosheets (SJN) that were not grafted with alkyl groups. While no pronounced change in the oil/water tension (~30 mN/m) was observed for the non-amphiphilic SJN particles, the CSAJN particles reduced the interfacial tension to ~17 mN/m. The frequency sweep measurements performed at a small strain amplitude (1%) with an interfacial cell and a biconical measuring system demonstrated an enhanced shear viscosity in presence of CSAJN particles (~1000 mN·s·m^−1^) compared to that of bare oil/water interface (~1 mN·s·m^−1^). Increasing the shear rate to 2.5 rad/s resulted in a reduction of the shear viscosity followed by reaching a plateau value, which indicated a gradual disruption of the interfacial network. Utilizing only 0.005 wt.% of the CSAJN particles in nanofluid flooding exhibited an improvement in the oil recovery efficiency by 18.31%, which was attributed to the formation of an elastic interfacial film at the oil/water interface and film climbing schematically shown in Figure 7d.

Not only the Janus character and amphiphilicity of the particle affect the rheological properties of the interfacially-trapped particle monolayer, but an applied shear flow itself is shown to impact the configuration of particles and their assembly, yielding interesting structural motifs, which, in turn, can be used to tune the rheological properties (i.e., shear viscosity). Studies have been carried out for assembly both at fluid interfaces and in bulk [259,260,261,262,263]; we will focus on the former here. Using a multicomponent Lattice-Boltzmann method, Rezvantalab et al. studied the directed assembly of a cluster of randomly oriented spherical Janus particles into ordered structures at a sheared interface between two immiscible fluids, schematically shown in Figure 7e [258]. Irrespective of the particle size and for an intermediate surface coverage (32–65%), the capillary-induced interactions resulting from the overlap of the interface deformations under shear flow yielded particle chain formation normal to the shear direction. Even after removing the flow field, the particle chains remained intact and particles only rotated to an upright orientation. Along with chemical anisotropy, particle geometry plays a role in the shear response of particles at fluid interfaces. Using molecular dynamics simulations, Rezvantalab et al. studied the rotational dynamics of single spherical, cylindrical, and disc-shaped Janus particles at fluid/fluid interfaces and demonstrated that depending on the particle shape, degree of amphiphilicity, and shear rate two modes of rotational dynamics exist, i.e., smooth tilt vs. tumbling [264]. However, irrespective of the particle dynamics, a steady-state orientation was achieved at the interface via the balance between shear-induced and capillary-induced torques; therefore, controlling the shear rate and surface chemistry for any particle geometry was suggested as a possibility for achieving a wide range of orientations at the interface and creating functional assemblies of Janus particles with tunable properties.

Shear-induced assembly behavior of Janus particles has also been examined at the interface between two polymer phases. Paiva et al. [265] studied the influence of shear flow on the directed assembly of Janus nanorods at the interface between two different polymer phases using Dissipative Particle Dynamics (DPD) technique and illustrated that aggregates of Janus nanorods can be trapped in unusual and counterintuitive configurations. It was shown that at shear rates high enough to overcome capillary torques, Janus rods move beyond basic tilting and exhibit tumbling behavior. The resulting structures were in the form of either Janus antiparallel configuration or stacked aggregate sheets such that favorable Janus interactions were enthalpically preferred. Wang et al. examined the correlation between microscopic morphologies of poly vinylidene fluoride/poly L-lactide (PVDF/PLLA) interface and the resulting linear rheological properties, and showed that systems compatibilized with Janus nanoparticles 110 nm in diameter silica particles grafted with PLLA/PMMA exhibit a prominently elevated elastic modulus, reduced interfacial tension, and retarded form relaxation of PVDF droplets. In addition, higher enhancement of dynamic moduli was reported, when compared to homogenous particles of equal loadings [74]. The solid-like behavior of Janus particle-filled blends was attributed to the orderly arrangement of Janus particles at the polymer/polymer interface and the molecular entanglement between the grafted long tails of Janus particles with the molecular chains of the respective polymer. Therefore, the presence of Janus particles at the interface not only promoted strong interfacial interactions between phases, but also led to the formation of a unique particle−polymer hybrid network, termed as “heterogeneous network” by the authors.

## 4. Concluding Remarks

Interfacial systems composed of fluids and surface-active materials have a wide range of applications in drug delivery, food science, personal care products, and in the chemical industry. These systems most often include surfactant molecules and/or colloidal particles. Janus particles are believed to render highly stable emulsions and foams when compared to their homogeneous counterparts. This article was aimed at reviewing the knowledge gained in the field on the role of Janus character in stability of interfacial systems by considering the following questions: What is the impact of particle properties (wetting, amphiphilicity, anisotropy) on the microstructure of the interface and its rheology? What is the connection between interfacial rheology and dynamic response of emulsions and foams? Main particle attributes that are shown to affect the stability of particle-laden interfaces were discussed, and the key parameters that contribute to the performance of particles at interfaces, both in static conditions and under flow, were reviewed. The role of factors unique to Janus particles, such as Janus balance and configuration at the interface, as well as their consequences for interfacial activity, interparticle-interactions, and response to applied stresses were highlighted. A number of studies examining the interfacial rheology of particle-laden interfaces, using both homogeneous and Janus particles, were discussed in order to review the central factors contributing to the resultant rheological properties and the presence (or lack thereof) of identified connections with the performance of interfacial systems.

With this knowledge available on the rheology of particle-laden interfaces, can we harness the Janus character in engineering interfacial systems with properties tailored to a specific application? Despite the evidence that Janus systems can lead to advances in industry, some gaps in knowledge remain on the relationship between rheological impact and interfacial stability caused by Janus particles. For instance, there are few papers on experiments carried out in an attempt to connect how the shear properties affect the interfacial stability and vice versa; even less is known on shear rheology of interfaces decorated with Janus particles. Rheology studies of interfacial Janus monolayers made with particles of different characteristics (such as Janus balance and amphiphilicity) comparing the resulting shear rheology at similar surface pressure or surface coverage are in order. Surface roughness, which is shown to modify the bulk rheology of particle suspensions, is another parameter that needs to be further investigated from the standpoint of interfacial rheology. In addition, a plethora of possibilities exist for the coupling of shape and chemical anisotropy. Design rules that can guide this choice would be very beneficial.

Probing the colloidal interactions on fluid interfaces has been a hot topic for recent investigations including studies on assembly of shape anisotropic and surface anisotropic (Janus) particles. While suggestions have been made to alter the stability of particle-stabilized emulsions using surfactants, a critical knowledge gap exists in complex interfacial systems comprised of colloidal particles and surfactant molecules, especially in elucidating the rich physical mechanisms that affect the synergism of heterogeneous particles and surfactants at interfaces and under an applied stress. Therefore, designing experiments and computations to understand the fundamental interactions at interfaces with particulate systems will advance the design of interfacial systems in applications involving flows. For example, designing Pickering foams suitable for hydraulic fracking requires our knowledge of the behavior of surfactants and particulate matter at fluid/fluid and fluid/solid interfaces, when the system is also subjected to a flow. Investigations on the synergistic effects of heterogeneous particles and surfactants on the stability and rheology of fluid/fluid interfaces are thus timely. With these connections established, we are better positioned to effectively exploit particle-stabilized systems tailored toward different industries.
